# Racial disparities and socioeconomic factors associated with post-acute rehabilitation facility utilization among Nevadans with Alzheimer’s disease and related disorders and extremity fractures: insights of age-friendly and dementia-friendly state planning in U.S.

**DOI:** 10.3389/fpubh.2024.1322830

**Published:** 2024-12-02

**Authors:** Sung Soo Oh, Ji Won Yoo, Stefan Chaudhry, Connor Jeong, Deanna Bae, Sumegha Mohanadasan, Leora Frimer, Yonsu Kim, Jay J. Shen

**Affiliations:** ^1^Department of Occupational and Environmental Medicine, Wonju College of Medicine, Yonsei University, Wonju, Republic of Korea; ^2^Department of Internal Medicine, Kirk Kerkorian School of Medicine at University of Nevada, Las Vegas, Las Vegas, NV, United States; ^3^Department of Biology, Stanford University, Palo Alto, CA, United States; ^4^The Connection Sphere, Las Vegas, NV, United States; ^5^Department of Healthcare Administration and Policy, School of Public Health, University of Nevada, Las Vegas, Las Vegas, NV, United States; ^6^Center for Health Disparities Research, University of Nevada, Las Vegas, Las Vegas, NV, United States

**Keywords:** Alzheimer’s disease, age-friendly, dementia-friendly, fracture, health disparity, post-acute care, rehabilitation facilities

## Abstract

**Background:**

Falls and extremity fractures often occur in people living with Alzheimer’s disease and related disorders (ADRD). In post-fracture care, these patients are cared for either at rehabilitation facilities or their homes. The coronavirus disease 2019 (COVID-19) pandemic limited the utilization of rehabilitation facilities. In areas with provider shortages, this trend poses a risk of disability and caregiver burdens, particularly in racial minorities who under-utilize rehabilitation facilities.

**Objective:**

To assess racial disparities in post-acute care (PAC) at rehabilitation facilities among people living with ADRD and extremity fractures during the COVID-19 pandemic.

**Methods:**

We summarized the PAC locations by (1) rehabilitation facilities (skilled nursing facilities and inpatient rehabilitation facilities) and (2) homes (homes with self-care and homes with services) for each study year. We observed the yearly percentage trends in PAC at rehabilitation facilities over the total post-acute discharge period. We assessed demographics (age, sex, and race), clinical comorbidities (fracture sites), utilization factors (pay source and hospital location), and COVID-19 pandemic status (pre−/post-pandemic years). We used multivariate logistic regression to estimate the association between these factors and PAC in the rehabilitation facilities.

**Results:**

The proportion of individuals receiving PAC declined in rehabilitation facilities, whereas the proportion of individuals receiving PAC at home with services continuously increased. Being Hispanic, presence of cerebrovascular disorder (CVD), use of Medicaid services, and the COVID-19 pandemic were associated with lower probabilities of utilizing rehabilitation facilities.

**Conclusion:**

Among the individuals with ADRD and extremity fractures, the proportion of those who underutilized rehabilitation facilities was higher in Hispanics compared with other races. Caregiving for Hispanics, presence of CVD, and use of Medicaid services were associated with the risk of disability and caregiver burden, due to shifting trends from rehabilitation facilities to homes with services. Geriatric workforce education should be prioritized to enhance the competencies of healthcare providers serving these individuals to relieve caregiver burdens in areas with provider shortage.

## Graphical abstract

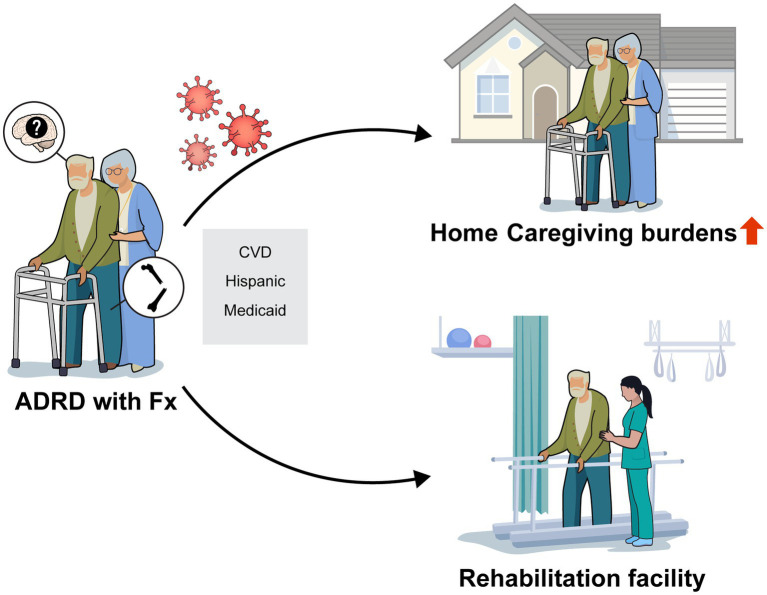

Utilization of rehabilitation facilities during the COVID of ADRD with fracture.

## 1 Introduction

More than 6 million people are living with Alzheimer’s disease and related disorders (ADRD) in the United States (US) (1). In the recent Agency for Healthcare Research and Quality systematic review, the overwhelming majority were disconnected from the delivery of system care for ADRD, which then led to low-value care with high burdens of healthcare and societal costs (2). The estimated annual cost of Medicare beneficiaries with ADRD (USD 43,644) was approximately three times the cost of those without ADRD (USD 14,660) in 2023 (1). People living with ADRD have been reported to be at high risks of falls, fractures, disability, and long-term facility stay, compared with those without ADRD (3). Living with ADRD increases post-acute care (PAC) requirements for recovering from extremity fractures because of the difficulty in following rehabilitation and precaution instructions related to communication challenges and behavioral symptoms (4–7). The coronavirus disease 2019 (COVID-19) pandemic has changed the delivery landscape of PAC utilization (8). Long-term care (LTC) and skilled nursing facilities (SNFs) for Medicare beneficiaries have been associated with adverse health outcomes, such as increased mortality and limited logistics due to decreased transfer between acute care hospitals and SNFs (8). These trends may result in spillover effects, such as unpaid dementia caregiver’s emotional distress and negative mental and physical health outcomes—monetary values triggered by the COVID-19 pandemic (1). These effects are expected in socially disadvantaged populations, such as Hispanics or Medicaid beneficiaries, who are known to underutilize SNFs for PAC, compared with non-Hispanic Whites or Medicare beneficiaries (9–12). The state of Nevada has the third fastest growing incidence rate of ADRD and the highest growing rate of ADRD-related health care expenditures in the U.S. (1). Similar to traditional provider shortage states sharing similar demographics (a population range of 3–5 million and larger surface area of rural areas), the State of Nevada had the fewest primary care providers *per capita* in the U.S. (13). Caregiving burdens in unpaid family members or other caregivers of people living with ADRD may be triggered when the extremity fracture recovery process occurs at home instead of an SNF in a provider shortage area, State of Nevada. Therefore, we aimed to evaluate racial disparities and socioeconomic factors associated with the PAC utilization at rehabilitation facilities among people with ADRD and extremity fractures in the State of Nevada. Thus, our examination provides the workforce education and policy-making insights of planning the establishment of an age-friendly and dementia-friendly state in a provider shortage area.

## 2 Materials and methods

### 2.1 Data source and study population

The publicly available State Inpatient Database (SID) was used. The SID contains more than 95% of the hospital discharge information from all community hospitals in the participating states. The SID was originally developed for the Healthcare Cost and Utilization Project (HCUP) by the Agency for Healthcare Research and Quality (14). The SID includes de-identified patient-level information on demographics, diagnostic and procedure codes, and discharge location (14). The Nevada SID files were constructed from hospital discharge files received from the University of Nevada, Las Vegas (UNLV) and the Center for Health Information Analysis (CHIA) under the authority of the Nevada Division of Healthcare Financing and Policy (14). The CHIA provided the HCUP with inpatient data from acute-care general, specialty, and rehabilitation hospitals in Nevada. The study period was from 2018 to 2021. The number of participating hospitals was 50, and the total number of discharged patients was approximately 360,000 annually. Among them, 24,532 patients aged 65 years or older were discharged from the hospital after being admitted for upper and lower extremity fractures. The number of patients with ADRD was 4,310. We identified extremity fractures and ADRD using the International Classification of Diseases, 10th revision, Clinical Modification (ICD-10-CM), as shown in Supplementary Table 1 (15, 16).

### 2.2 Measured outcomes and variables

The measured outcome was PAC location after extremity fractures: rehabilitation facilities (SNFs and inpatient rehabilitation facilities) and homes (homes with self-care and homes with services) in each year of the study. We excluded less than 1% of patients discharged, including those who used other intermediate care facilities and those who left against medical advice. We evaluated trends in PAC in rehabilitation facilities and homes over the total PAC discharge period. We measured patient-level characteristics including demographics (age, sex, and race), clinical factors (comorbidities and fracture locations), utilization factors (pay source and hospital location), and COVID-19 pandemic status (pre−/during pandemic years). Choice of the above comorbidities was relevant to previous literature related to either extremity fractures or discharge to PAC (3, 4, 6, 7, 10). Pre-COVID-19 was defined as the period from January 2018 to December 2019, and post-COVID-19 was defined as the period from January 2020 to December 2021. Age was divided into three categories: 65–74 years, 75–84 years, and ≥ 85 years. Race was classified as non-Hispanic White person or Black person; Hispanic; Asian, Hawaiian, and Pacific Islander (AHPI); and others. Pay sources were divided into four groups: Medicare, Medicaid, private insurance, and other insurance services and self-payments. “RL_RUCC” variable contained a uniform code for hospital location and was divided into metro/urban (1–7) and rural (8, 9) areas (17). Rural–Urban Continuum Codes (RUCC) subdivides counties into 10 categories distinguished by population size in census-defined urbanized areas and by adjacency to metropolitan areas. To be adjacent, counties must be contiguous and have at least 2% of the resident labor force commuting to a central metropolitan county. A county-based system such as RUCC, which attempts to describe the diversity in settlement patterns in a relatively large area by a single number, may not provide an accurate depiction. However, because county boundaries do not change much, every county will be represented by a measure, even after an extended period of time. RUCC was developed in the U.S. Department of Agriculture’s Economic Research Service, as a refinement of the Office of Management and Budget (OMB) Metropolitan Statistical Area (MSA) definition (17). Bone mineral disorder, cerebrovascular disease (CVD), and substance use disorder (SUD) were assessed as comorbidities of extremity fracture (3, 4, 6, 7, 10). Extremity fracture location was divided into upper and lower extremities. The codes for each condition were selected accordingly (Supplementary Table 1).

### 2.3 Statistical analysis

Bivariate analysis with Pearson’s chi-square test was used to compare demographics, clinical factors, and utilization factors by race. Multivariate regression analysis was conducted to evaluate factors affecting PAC at rehabilitation facilities. Estimation was performed using odds ratios (OR) and the corresponding 95% confidence intervals (CIs). Analyses were adjusted for all covariates, and two-sided *p* < 0.05 was considered statistically significant. Analyses were performed using the SAS software, version 9.4 (SAS Institute, Cary, NC, USA) (18). As the Nevada SID database provides administrative de-identified data, the requirement of Institutional Review Board approval and written informed consent was waived by the ethics committee of the UNLV (IRB no. 1098939-3).

## 3 Results

Non-Hispanic White persons had the highest proportion of male individuals, while AHPIs had the highest proportion of female individuals. Non-Hispanic White persons and Black persons had the highest proportion of those who used Medicare (90%), while Hispanics had the highest proportion of those who used Medicaid. Private insurance was commonly reported among AHPIs. Regarding residential areas, the proportion of Black persons living in urban areas was the highest (approximately 96%), while the proportion of White persons living in rural areas was relatively high compared with other races. CVD rates were higher among Black persons than among other races. SUD was relatively lower in Hispanics and Asians, compared with White persons and Black persons (Table 1).

**Table 1:** Descriptive analysis of demographics, clinical factors, and utilization factors by race groups (N, %).

	Non-Hispanic Whites *n* = 3,403	Blacks *n* = 198	Hispanics *n* = 240	AHPI *n* = 190	Other races *n* = 279	*p*-value
Demographics						
Age (mean, standard deviation)	82.80 (6.68)	81.30 (7.18)	82.90 (6.32)	84.53 (5.60)	82.49 (7.30)	**<0.05**
Gender						
Male (*n* = 1,346)	1,101 (32.35)	54 (27.27)	60 (25.00)	39 (20.53)	92 (32.97)	**<0.05**
Female (*n* = 2,964)	2,302 (67.65)	144 (72.73)	180 (75.00)	151 (79.47)	187 (67.03)	
Clinical factors						
BMD (*n* = 715)	546 (16.04)	30 (15.15)	53 (22.08)	35 (18.42)	51 (18.28)	0.12
CVD (*n* = 311)	238 (6.99)	30 (15.15)	11 (4.58)	13 (6.84)	19 (6.81)	**<0.05**
SUD (*n* = 444)	372 (10.93)	22 (11.11)	11 (4.58)	8 (4.21)	31 (11.11)	**<0.05**
Utilization factors						
Pay source						
Medicare (*n* = 3,879)	3,084 (90.63)	179 (90.40)	206 (85.83)	164 (86.32)	246 (88.17)	**<0.05**
Medicaid (*n* = 27)	11 (0.32)	0 (0.00)	12 (5.00)	0 (0.00)	4 (1.43)	
Private (*n* = 266)	197 (5.79)	11 (5.56)	15 (6.25)	19 (10.00)	24 (8.60)	
Other (*n* = 138)	111 (3.26)	8 (4.04)	7 (2.92)	7 (3.68)	5 (1.79)	
Hospital location						
Metro/urban (*n* = 3,548)	2,753 (80.90)	191 (96.46)	213 (88.75)	166 (87.37)	225 (80.65)	**<0.05**
Rural (*n* = 762)	650 (19.10)	7 (3.54)	27 (11.25)	24 (12.63)	54 (19.35)	
PAC locations						
Rehabilitation facilities						
SNF (*n* = 2,104)	1730 (61.52)	84 (48.55)	94 (45.63)	80 (49.38)	116 (50.43)	**<0.05**
IRF (*n* = 536)	407 (14.47)	32 (18.50)	30 (14.56)	27 (16.67)	40 (17.39)	
Home						
Self-care (*n* = 304)	229 (8.14)	18 (10.40)	23 (11.17)	15 (9.26)	19 (8.26)	
With services (*n* = 639)	446 (15.86)	39 (22.54)	59 (28.64)	40 (24.69)	55 (23.91)	

AHPI, Asian, Hawaiian, Pacific Islanders; BMD, bone metabolic disorder; CVD, cerebrovascular disease; IRF, inpatient rehabilitation facilities; PAC, post-acute care; SNF, skilled nursing facilities; SUD, substance use disorder. Bolded *p*-value is statistically significant.

**Figure 1:**
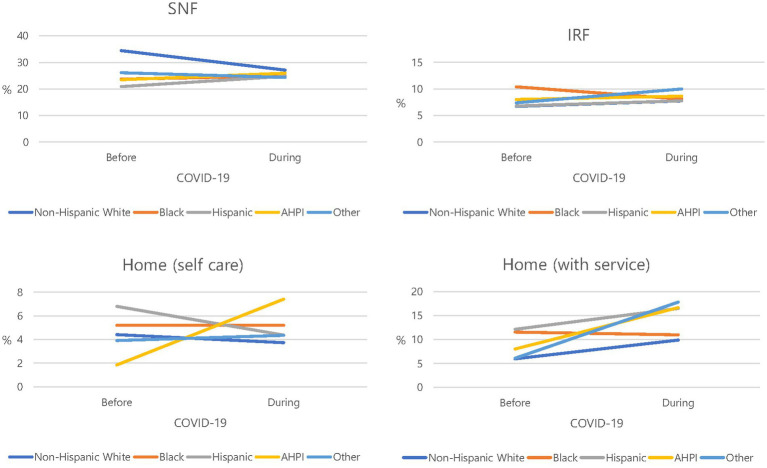
Trends of the percentage of utilizing rehabilitation facilities by race.

PAC at rehabilitation facilities decreased overall since the COVID-19 pandemic. The decrease was the greatest in non-Hispanics White persons (Figure 1 and Table 2). Table 3 presents regression results, predictors of PAC utilization at rehabilitation facilities by COVID -19 pandemic, demographics, and clinical and utilization factors. COVID pandemic was associated with lower probability of utilizing facilities as PAC locations (OR 0.771, 95% CI 0.668 to 0.890, *p* < 0.001). Approximately 43% fewer Hispanics than non-Hispanics (White persons) were transferred to a PAC location (*p* = 0.002). More patients with lower extremity fractures were transferred for PAC (*p* < 0.001). The patients with CVD as a comorbidity were approximately 24% less likely to be transferred (*p* = 0.030). Medicaid beneficiaries were approximately 60% less likely than private insurers to be transferred to a PAC location (*p* = 0.031).

**Table 2:** Comparison of post-acute care locations before and during COVID-19 pandemic by race (N, %).

	Non-Hispanic Whites *n* = 3,403		Blacks *n* = 198		Hispanics *n* = 240		AHPI *n* = 190		Other races *n* = 279	
	Before	During	Before	During	Before	During	Before	During	Before	During
PAC locations										
Rehabilitation facilities										
SNF (*n* = 2,104)	968 (34.42)	762 (27.10)	41 (23.70)	43 (24.86)	43 (20.87)	51 (24.76)	38 (23.46)	42 (25.93)	60 (26.09)	56 (24.35)
IRF (*n* = 536)	189 (6.72)	218 (7.75)	18 (10.40)	14 (8.09)	14 (6.80)	16 (7.77)	13 (8.02)	14 (8.64)	17 (7.39)	23 (10.00)
Home										
Self-care (*n* = 304)	124 (4.41)	105 (3.73)	9 (5.20)	9 (5.20)	14 (6.80)	9 (4.37)	3 (1.85)	12 (7.41)	9 (3.91)	10 (4.35)
With services (*n* = 639)	168 (5.97)	278 (9.89)	20 (11.56)	19 (10.98)	25 (12.14)	34 (16.50)	13 (8.02)	27 (16.67)	14 (6.09)	41 (17.83)
***p*-value**	**<0.001**		0.914		0.506		0.118		**0.014**	

AHPI, Asian, Hawaiian, Pacific Islanders; IRF, inpatient rehabilitation facilities; PAC, post-acute care; SNF, skilled nursing facilities. Bolded *p*-value is statistically significant.

**Table 3:** Predictors of post-acute care at rehabilitation facilities by COVID-19 pandemic, demographics, clinical, and utilization factors.

	Referent	95% confidence intervals			
		Odds ratio	Lower limit	Upper limit	*p*-value
**COVID pandemic (2020, 2021)** ***n* = 1,897**	Before COVID (2018, 2019) *n* = 2,413	0.771	0.668	0.890	**<0.001**
Demographics					
Age	65–74 (*n* = 1,749)				
75–84 (*n* = 1,387)		1.153	0.927	1.434	0.234
85–90 (*n* = 1,174)		0.982	0.796	1.211	0.801
Gender	Male (*n* = 1,346)				
Female (*n* = 2,964)		1.042	0.907	1.198	0.592
Race	Non-Hispanic Whites (*n* = 3,403)				
Blacks (*n* = 198)		0.771	0.507	1.174	0.275
**Hispanics (*n* = 240)**		0.575	0.384	0.859	**0.002**
AHPI (*n* = 190)		0.982	0.602	1.601	0.097
Other races (*n* = 279)		1.011	0.677	1.512	0.070
Clinical factors					
Fracture location					
Upper extremity (*n* = 1,085)		1.065	0.722	1.576	0.524
**Lower extremity (*n* = 3,225)**		1.756	1.223	2.531	**<0.001**
Comorbidity					
BMD (*n* = 715)		0.965	0.813	1.146	0.674
**CVD (*n* = 311)**		0.761	0.598	0.968	**0.030**
SUD (*n* = 444)		1.219	0.980	1.516	0.076
Utilization factors					
Pay source	Private (*n* = 266)				
Medicare (*n* = 3,879)		1.267	0.984	1.631	0.066
**Medicaid (*n* = 27)**		0.405	0.170	0.966	**0.031**
Other (*n* = 138)		1.417	0.902	2.225	0.120
Hospital location	Metro/urban (*n* = 3,548)				
Rural (*n* = 762)		1.008	0.852	1.193	0.841

AHPI, Asian, Hawaiian, Pacific Islanders; BMD, bone metabolic disorder; CVD, cerebrovascular disease; IRF, inpatient rehabilitation facilities; PAC, post-acute care; SNF, skilled nursing facilities; SUD, substance use disorder. Bolded *p*-value is statistically significant.

## 4 Discussion

A decline of more than 20% in the utilization rate of rehabilitation facilities during the COVID-19 pandemic was observed across all racial groups in our study. This trend has been observed in other studies on limiting SNF transition during the COVID-19 pandemic (19). Although the association was statistically marginal, the COVID-19 pandemic triggered the limitation of rehabilitation facility utilization by the AHPI populations. Traditionally, for example, Native Hawaiians have been heavily relying on female caregivers and home-oriented caregiving from their cultural context of underutilizing facilities at PAC locations (20). Reports of racist and xenophobic incidents directed toward persons perceived to be of Asian descent, especially older adults, increased (21, 22). In our study, both being Hispanic and a Medicaid beneficiary played dual roles in the underutilization of rehabilitation facilities. Moreover, Hispanics were more likely to be Medicaid beneficiaries compared to non-Hispanic white counterparts. This pattern has also been observed in other studies that assessed SNF utilization patterns (9–12, 23). A study has also revealed that Hispanics are less likely to have access to high-rated Medicare Advantage (MA) plans and are more likely to shift to either low-rate MA plans or Medicaid enrollment (24). Along with Hispanics’ strong family and social ties, Hispanics have been reported as their fewer financial resources account for disparity of rehabilitation facilities utilization (12, 25). However, the interpretation of this shift is largely unclear and requires further investigation.

In this study, the PAC transition rate was high among patients with lower-extremity fractures. This is because the part that has the most direct effect on activities of daily living is the lower extremity. Therefore, it is thought that patients with lower extremity fractures with functionally restricted movement will undergo more PAC transitions to rehabilitation facilities than to their homes. In addition, because the severity is likely to increase, PAC transition rates are expected to increase. Those with CVD underutilized rehabilitation facilities in this study. It is speculated that rehabilitation potential is lower when stroke and its sequelae add to the burden of extremity fractures. The burdens of managing both conditions increase the risk of disability and caregiver burden after discharge from the hospital. This finding highlights the importance of timely and coordinated care, in this case, using a multifaceted and innovative home/community-based approach, such as the Guiding an Improved Dementia Experience (GUIDE) model (26). The innovative GUIDE model delivers on the Biden Administration’s April 2023 Executive Order 14095 by advancing access equity of the underserved communities, racial and ethnic minorities’ ADRD caregivers and enhancing equal access, especially, home and community-based care services (26). The number of beds in nursing homes, including LTC facilities and SNFs, in the US has decreased by approximately 25% over the past decade with the increase in the availability of home-and community-based services (27). This trend of decline in the number of nursing home beds has worsened since the beginning of the COVID-19 pandemic. However, the supply of these services still lags behind the demand (27). The lack of access to SNFs, particularly among racial and ethnic minorities and Medicaid beneficiaries, may lead to the need for more complex care in individuals at a greater risk of adverse outcomes, caregiving burdens related to hospitalizations, and responsive increases in healthcare costs among people living with ADRD (1, 4). These findings highlight the importance of educating the geriatric healthcare workforce that serves socially disadvantaged populations, Hispanics, and Medicaid beneficiaries, to mitigate concentrated caregiving burdens (16). Cultural and linguistic sensitivity geriatric workforce training includes familism, language, literacy, older adult justice, and logistical barriers (28). Collaborative primary care for individuals with ADRD, Healthy Aging Brain Center, demonstrated improved care coordination and resulted in producing net savings by reducing unnecessary hospitalization and ED visits due to caregiver burdens (29). Adult day care center-based virtual training for low-income ADRD caregivers may enhance the capacity of coping skills of caring for those with limited physical function (30). By promoting coordination of care planning with ADRD caregivers, primary telehealth may avoid unnecessary hospitalizations or emergency department visits of ADRD individuals (31). The evidence-based Age-Friendly Health System frameworks, 4 M (what matters, mobility, medication, and mentation), has been applied for training the geriatric healthcare workforce effectively, and it is locally adaptable, especially for racial and ethnic minority older adults in the State of Nevada (31–33). In addition, telehealth as primary care has been delivered to people living with ADRD in areas with provider shortages and has achieved more efficient care coordination by reducing healthcare costs by 20% (31). Planning strategies of establishing age-friendly and dementia-friendly states are prioritized to the workforce capacity enhancement and innovative access to care development (i.e., telehealth) that is more practical to accomplish rather than structural investment (i.e., increase of hospital beds) in a provider shortage area like the State of Nevada (32, 34). Our study has a great advantage in that a representative national database, the SID, was used. In addition, this is the first study to be conducted on PAC transition in patients with ADRD and fractures. However, this study has some limitations. First, the number of people living with ADRD may have been underreported in the SID. For example, the ADRD diagnosis rate is low in acute hospital care due to a lack of interoperability in outpatient care, and cognitive function screening has been under-implemented in provider shortage areas. Second, the ADRD degree was not determined; controlling for the ADRD degree may have helped to understand the dynamics of PAC transition after extremity fracture. An imbalance in the sample size of racial minorities may have influenced the statistical significance of rehabilitation facility utilization. Another limitation of this study was the lack of information on community resources and caregiver availability, limiting the interpretation of PAC location decisions. Therefore, our analysis is preliminary until additional, more representative data are analyzed to confirm our findings.

## 5 Conclusion

Among the individuals with ADRD and extremity fractures in this study, the rate of underutilization of rehabilitation facilities was higher among Hispanics than among people of other races. The COVID-19 pandemic limited the utilization of rehabilitation facilities by more than 20%. Caregiving for Hispanics, presence of CVD, and use of Medicaid services were associated with the risk of disability and caregiver burden, due to shifting trends from rehabilitation facilities to homes with services. Geriatric healthcare workforce education should be prioritized to enhance the competencies of healthcare providers serving these individuals, to relieve caregiver burdens in provider shortage areas.

## Data Availability

The original contributions presented in the study are included in the article/Supplementary material, further inquiries can be directed to the corresponding author.
