# Supplementary material for: Racial disparities and socioeconomic factors associated with post-acute rehabilitation facility utilization among Nevadans with Alzheimer’s disease and related disorders and extremity fractures: insights of age-friendly and dementia-friendly state planning in U.S
Source: Front Public Health. 2024 Dec 2;12:1322830. doi: 10.3389/fpubh.2024.1322830 (PMC11647528; doi:10.3389/fpubh.2024.1322830)
Supplement: Supplementary file 1 [file Table_1.DOCX]

Supplement Table 1. ICD-10-CM codes

| **ICD-10-CM code** | **Full name** |
| --- | --- |
| **ADRD** |  |
| F01 | Vascular dementia (unspecified severity) |
| F02 | Dementia in other diseases classified elsewhere |
| F03 | Unspecified dementia |
| **Comobidities** |  |
| **BMD**  **(bone metabolic disease)** |  |
| M80 | Osteoporosis (with current pathological fracture) |
| M81 | Osteoporosis (without current pathological fracture) |
| M83 | Adult osteomalacia |
| M84 | Disorder of continuity of bone |
| M85 | Other disorders of bone density and structure |
| **CVD**  **(Cerebrovascular diseases)** |  |
| I60 | Nontraumatic subarachnoid hemorrhage |
| I61 | Nontraumatic intracerebral hemorrhage |
| I62 | Other and unspecified nontraumatic intracranial hemorrhage |
| I63 | Cerebral infarction |
| I65 | Occlusion and stenosis of precerebral arteries, not resulting in cerebral infarction |
| I66 | Occlusion and stenosis of cerebral arteries, not resulting in cerebral infarction |
| I67 | Other cerebrovascular diseases |
| I68 | Cerebrovascular disorders in diseases classified elsewhere |
| I69 | Sequelae of cerebrovascular disease |
| **Substance use** |  |
| F10 | Alcohol related disorders |
| F11 | Opioid related disorders |
| F12 | Canabis related disorders |
| F13 | Sedative, hypnotic, or anxiolytic related disorders |
| F14 | Cocaine related disorders |
| F15 | Other stimulant related disorders |
| F16 | Hallucinogen related disorders |
| F17 | Nicotine dependence |
| F18 | Inhalant related disorders |
| F19 | Other psychoactive substance related disorders |
| Z72.0 | Tobacco use |
| **Upper extremity fracture** |  |
| **Shoulder/ Humerus shaft** |  |
| S42.0 | Fracture of clavicle |
| S42.1 | Fracture of scapula |
| S42.2 | Fracture of upper end of humerus |
| S42.3 | Fracture of shaft of humerus |
| S42.9 | Fracture of shoulder girdle, part unspecified (Fracture of shoulder NOS) |
| **Elbow** |  |
| S42.4 | Fracture of lower end of humerus |
| S52.0 | Fracture of upper end of ulna |
| S52.1 | Fracture of upper end of radius |
| **Radius, Ulna shaft, Wrist** |  |
| S52.2 | Fracture of shaft of ulna |
| S52.3 | Fracture of shaft of radius |
| S52.9 | Unspecified fracture of forearm |
| S52.5 | Fracture of lower end of radius |
| S52.6 | Fracture of lower end of ulna |
| **Carpal bone/ Hand** |  |
| S62.0 | Fracture of navicular (scaphoid) bone of wrist |
| S62.1 | Fracture of other and unspecified carpal bone(s) |
| S62.2 | Fracture of first metacarpal bone |
| S62.3 | Fracture of other and unspecified metacarpal bone |
| S62.6 | Fracture of other and unspecified finger(s) |
| S62.9 | Unspecified fracture of wrist and hand |
| **Lower extremity fracture** |  |
| **Hip/ Femur shaft** |  |
| S72.0 | Fracture of head and neck of femur |
| S72.1 | Petrochanteric fracture |
| S72.2 | Subtrochanteric fracture of femur |
| S72.3 | Fracture of shaft of femur |
| S72.8 | Other fracture of femur |
| S72.9 | Unspecified fracture of femur |
| **Knee/ Patella** |  |
| S72.4 | Fracture of lower end of femur |
| S82.0 | Fracture of patella |
| S82.1 | Fracture of upper end of tibia |
| S82.81 | Torus fracture of upper end of fibula |
| **Tibia, Fibula shaft/ Ankle** |  |
| S82.2 | Fracture of shaft of tibia |
| S82.3 | Fracture of lower end of tibia |
| S82.4 | Fracture of shaft of fibula |
| S82.5 | Fracture of medial malleolus |
| S82.6 | Fracture of lateral malleolus |
| S82.82 | Torus fracture of lower end of fibula |
| S82.84 | Bimalleolar fracture of lower leg |
| S82.85 | Trimalleolar fracture of lower leg |
| S82.86 | Maisonneuve’s fracture |
| S82.87 | Pilon fracture of tibia |
| S82.89 | Other fractures of lower leg |
| **Tarsal bones/ Foot** |  |
| S92.0 | Fracture of calcaneus |
| S92.1 | Fracture of talus |
| S92.2 | Fracture of other and unspecified tarsal bone(s) |
| S92.3 | Fracture of metatarsal bone(s) |
| S92.4 | Fracture of great toe |
| S92.5 | Fracture of lesser toe(s) |
| S92.9 | Unspecified fracture of foot and toe |
